# Temocillin use as a carbapenem-sparing option in a UK teaching hospital for treating serious Gram-negative bacterial infections

**DOI:** 10.1093/jacamr/dlac111

**Published:** 2022-10-31

**Authors:** D A Enoch, M E Murphy, T Gouliouris, R Santos, C Micallef

**Affiliations:** Clinical Microbiology & Public Health Laboratory, UK Health Security Agency, Addenbrooke’s Hospital, Hills Road, Cambridge CB2 OQW, UK; Department of Microbiology, UKNHS GGC Glasgow Royal Infirmary, New Lister Building, Alexandra Parade, Glasgow G31 2ER, UK; Clinical Microbiology & Public Health Laboratory, UK Health Security Agency, Addenbrooke’s Hospital, Hills Road, Cambridge CB2 OQW, UK; Department of Pharmacy, Addenbrooke’s Hospital, Hills Road, Cambridge CB2 OQQ, UK; Department of Pharmacy, Addenbrooke’s Hospital, Hills Road, Cambridge CB2 OQQ, UK

We read the article about temocillin for the treatment of invasive Enterobacterales infections with interest.^1^ Temocillin is a narrow-spectrum penicillin with activity against ESBL-producing Enterobacterales (ESBL-PE). It could be used in place of carbapenems for some infections due to these infections (i.e. bacteraemia, pneumonia, urinary tract infection but not meningitis).^2^ This option is becoming increasingly important in the era of emerging carbapenem resistance. Temocillin has also been shown to have less of an impact on the intestinal microbiota than cephalosporins^3^ or piperacillin/tazobactam.^4^

We sought to determine the appropriateness, effectiveness and tolerability of temocillin prescribing in Cambridge University Hospitals (CUH) NHS Foundation Trust. This was considered a service evaluation and ethical approval was not required.

We performed a single-centre retrospective data review of all adult inpatients who received temocillin between 1 October 2016 and 31 July 2017 at CUH. Temocillin was approved for use in CUH when a urine or blood culture isolate was confirmed to be an ESBL-PE and susceptible to temocillin (by BSAC methodology) and resistant to oral agents, in order to preserve carbapenems and piperacillin/tazobactam. Its use was therefore restricted to be only recommended by a microbiology consultant. As part of the formulary submission we were required to analyse its use to confirm it was used appropriately. It was used as monotherapy unless the microbiologist was concerned about polymicrobial infection.

Epidemiological data, duration of therapy and duration of carbapenem-sparing were recorded and analysed. Temocillin was dosed at 4 g/day (2 g IV q12h), except in cases of reduced renal function where dosing followed local guidance.

A total of 24 patients (14 male; 58%) were included. Two patients required two courses of therapy (total 26 courses) due to recurrence (one due to inadequate source control). The age of the patients ranged from 23–96 years (mean 69 years). Sixteen patients were cared by medicine/medicine for elderly, four by urology and two each by transplant and neurosurgery (Table 1).

**Table 1.** Clinical features of patients receiving temocillin

Patient	New/recurrence	Age	Sex	Specialty	Sample	Source	Dose	GFR (mL/min)	Appropriate dose?	Duration of therapy (days)	Reason for stopping	Previous antibiotics	McCabe	CCMS	Albumin (g/L)	CRP (mg/L)	Peak temperature (°C)	Trends before switch	Outcome at 1 week	Outcome at discharge	Breakthrough infection	Organism
1	New	53	F	Renal transplant	blood	urinary	1 g twice daily	36	y	5	completion of course	piperacillin/tazobactam, meropenem	non-fatal	3	32	45	38.5	improving	improving	alive	y: source control	*E. coli*
1	Recurrence				blood	urinary	1 g twice daily	47	y	4	switch to ertapenem	meropenem	—	—	29	98	39.7	improving	improving	alive	n	*E. coli*
2	New	58	F	Renal transplant	blood	urinary	500 mg once daily	5	y	10	completion of course	piperacillin/tazobactam, meropenem	non-fatal	4	21	289	35.9	improving	improving	alive	n	*E. coli*
3	New	83	F	Medicine	blood	urinary	2 g twice daily	62	y	1	switch to ertapenem	co-amoxiclav, gentamicin	non-fatal	1	na	115	39.2	improving	improving	alive	n	*E. coli*
4	New	47	M	Medicine	blood	biliary	2 g twice daily	60	y	9	completion of course	meropenem	ultimately fatal	7	15	50	40.3	improving	improving	alive	n	*E. coli*
5	New	23	M	Medicine	blood	bowel	1 g twice daily	na	y	15	completion of course	piperacillin/tazobactam, meropenem	rapidly fatal	6	28	198	37.5	improving	improving	dead	n	*E. coli*
5	Recurrence				blood	bowel	1 g twice daily	na	y	10	died	piperacillin/tazobactam, meropenem	—	—	19	100	36.8	improving	improving	dead	n	*E. coli*
6	New	70	M	Medicine	blood	urinary	1 g once daily	50	y	2	switch to ertapenem	co-amoxiclav, meropenem	ultimately fatal	11	25	174	39.0	improving	improving	alive	n	*E. coli*
7	New	77	M	Medicine	blood	urinary	1 g once daily	20	y	9	completion of course	piperacillin/tazobactam, meropenem	ultimately fatal	6	36	93	39.1	improving	improving	alive	n	*E. coli*
8	New	87	F	DME	urine	urinary	1 g twice daily	80	no, underdose	2	switch to ertapenem	co-amoxiclav, piperacillin/tazobactam	non-fatal	1	25	120	38.6	improving	improving	alive	n	*E. coli*
9	New	96	F	neuro-surgery	blood	urinary	1 g twice daily	74	no, underdose	8	completion of course	piperacillin/tazobactam, meropenem	rapidly fatal	7	28	95	38.0	improving	improving	alive	n	*E. coli*
10	New	60	F	Urology	urine	urinary	1 g twice daily	72	no, underdose	4	switch to ertapenem	trimethoprim	non-fatal	2	na	18	36.0	improving	improving	alive	n	*E. coli*
11	New	69	M	Medicine	blood	urinary	2 g twice daily	76	y	4	switch to ertapenem	co-amoxiclav, meropenem	non-fatal	10	34	53	38.5	improving	improving	alive	n	*E. coli*
12	New	62	M	Urology	blood	urinary	1 g twice daily	57	y	2	switch to ertapenem	co-amoxiclav, piperacillin/tazobactam, amikacin, ciprofloxacin	non-fatal	3	19	49	38.7	improving	improving	alive	n	*E. coli*
13	New	80	M	Medicine	blood	HAP	1 g twice daily	53	y	6	completion of course	meropenem	non-fatal	3	na	70	37.8	improving	improving	alive	n	*E. coli*
14	New	74	M	Medicine	blood	urinary	1 g twice daily	59	y	9	completion of course	co-amoxiclav, meropenem	ultimately fatal	2	18	73	39.2	improving	improving	alive	n	*E. coli*
15	New	65	F	Medicine	blood	HAP	1 g twice daily	86	no, underdose	10	completion of course	co-amoxiclav, meropenem	ultimately fatal	2	na	64	37.0	improving	improving	alive	n	*E. coli*
16	New	94	M	DME	blood	urinary	1 g once daily	26	y	10	completion of course	piperacillin/tazobactam, meropenem	ultimately fatal	12	20	78	37.4	improving	improving	alive	n	*E. coli*
17	New	74	M	Urology	urine	urinary	1 g twice daily	20	n, overdose	3	switch to pivmecillinam	co-amoxiclav, gentamicin	ultimately fatal	8	20	50	38.9	improving	improving	alive	n	*E. coli*
18	New	87	M	Medicine	blood	HAP	2 g twice daily	63	y	6	completion of course	co-amoxiclav, meropenem	non-fatal	3	24	238	39.1	improving	improving	alive	n	*E. coli*
19	New	62	F	Medicine	blood	bowel	2 g twice daily	80	y	4	completion of course	piperacillin/tazobactam, meropenem	non-fatal	3	28	8	40.3	improving	improving	alive	n	*E. coli*
20	New	88	F	DME	previous ESBL	wound	1 g twice daily	46	y	3	completion of course	none	non-fatal	1	35	94	37.7	na	improving	alive	n	—
21	New	82	M	Medicine	previous ESBL	urinary	1 g twice daily	43	y	5	switch to ciprofloxacin	co-amoxiclav	non-fatal	2	na	114	40.1	na	improving	alive	n	—
22	New	51	M	Neuro-surgery	blood	urinary	2 g twice daily	218	y	3	completion of course	meropenem	non-fatal	2	30	30	39.7	improving	improving	alive	y: unknown	*E. coli*
23	New	74	F	Urology	blood	urinary	500 mg once daily	47	n, underdose	1	switch to ciprofloxacin	co-amoxiclav, gentamicin, meropenem	non-fatal	7	na	330	39.3	improving	improving	alive	n	*E. coli*
24	New	45	M	Medicine	blood	urinary	1 g twice daily	86	n, underdose	3	switch to meropenem	piperacillin/tazobactam, meropenem	non-fatal	5	22	98	40.2	declining	declining	alive	n	*E. coli*

CCMS, Charlson comorbidity score; CRP, C-reactive protein; DME, Department of Medicine for the Elderly; F, female; GFR, glomerular filtration rate; HAP, hospital-associated pneumonia; M, male; n, no; na, not applicable; y, yes.

Two patients (8%) had a rapidly fatal underlying condition, 7 (29%) had an ultimately fatal condition and 15 (63%) had a non-fatal underlying condition. The Charlson comorbidity score ranged from 1 to 12 (median 3). Three required intensive care.

Twenty-one episodes (81%) were bacteraemic; 19 of these were due to an ESBL-PE, whilst one had an AmpC-producing *Escherichia coli* and one had an *E. coli* with no ESBL or AmpC identified; three grew ESBL-PE in urine and two were commenced on temocillin as they had previously had ESBL-PE identified from urine/blood cultures.

Fourteen of 21 (67%) bacteraemic episodes were related to a urinary source, three (14%) had a bowel source (who received concomitant metronidazole), three (14%) had healthcare-associated pneumonia and one (5%) had cholangitis.

Duration of therapy varied between 1 and 15 days (mean 6 days). Reasons for stopping temocillin included completion of course (13; 50%), 7 (27%) episodes of switching to ertapenem to facilitate outpatient parenteral antimicrobial therapy, one patient died and 5 (19%) episodes had other explanations. None of the discontinuations were due to intolerance/toxicity and there were no reported side effects. Renal function at the time of commencing temocillin varied greatly during the study, with glomerular filtration rate ranging from 5 to 218 mL/min (median 53 mL/min). There was, therefore, a wide range of dosing given. Overall, 19 (73%) had the correct dose, with 6 being underdosed and one being overdosed.

Two patients (8%) had recurrence of disease and one patient died. Patients had received 0–37 g (median 4 g) of meropenem prior to switching. Twenty-five (96%) of the episodes were improving on their previous regimen prior to switching to temocillin; they showed improvement 1 week after switch and 24 (92%) of these episodes showed improvement 1 month after switch. One patient was deteriorating prior to switching to temocillin and continued to deteriorate. Source control and switching back to meropenem occurred in this patient. One hundred and forty-eight days of total carbapenem-sparing was achieved.

We provide data on the use of temocillin as a carbapenem-sparing agent in the management of serious ESBL-PE infections including 21 bacteraemic patients. Safe and effective alternatives are required in order to preserve carbapenems for seriously ill patients and prevent the emergence of resistance. Temocillin was used in patients with significant comorbidities. However, most patients had a urinary source and most patients were improving prior to switching to temocillin.

Data supporting the use of temocillin for treating bacteraemic patients is currently limited to one case–control study^5^ and three case series^1–3^ involving 138 patients in total (26, 42, 40 and 30, respectively).

We found that dosing was inadequate according to renal function in six of our patients. This requires improvement, as appropriate dosing was associated with improved outcome in another study.^2^

Important limitations include the single-centre study design involving a small number of patients with non-comparative data. In addition, the majority of patients were clinically and biochemically improving before antibiotics were switched to temocillin while awaiting susceptibility results.

Nevertheless, we believe that these data add to the current literature supporting the use of temocillin as follow-on therapy for treating serious infections, including bacteraemias, with limited treatment options, as a way to spare carbapenems. Further work is required, in the form of a prospective study or randomised control study to formally assess this further, such as that proposed by the ASTARTÉ trial.^6^
